# Leptin Alleviates Inflammatory Response in Myocardial Ischemia Reperfusion Injury

**DOI:** 10.1155/2022/8707061

**Published:** 2022-03-09

**Authors:** Shu Xu, Dengshun Tao

**Affiliations:** General Hospital of Northern Theater Command, Department of Cardiovascular Surgery, China

## Abstract

**Objective:**

To investigate the role of leptin in regulating cell inflammation and protecting myocardium after myocardial ischemia-reperfusion injury in rats through signaling pathway at tissue and molecular protein levels.

**Methods:**

Healthy female SD rats were randomly divided into 4 groups, which were sham, I/R group, leptin low-dose intervention group, and high-dose intervention group (40 *μ*g/kg and 80 *μ*g/kg, respectively). Cardiac hemodynamics, myocardial enzymology, inflammatory indices, and pathological changes were observed. Western blot was used to observe the expression of PI3K, AKT, and NF*κ*B protein by leptin.

**Results:**

Leptin can improve the hemodynamics of cardiac ischemia-reperfusion rats, improve the expression of myocardial enzymology, reduce the release of cardiac and serum inflammatory factors, increased PI3k, AKT, and NF*κ*B expression, and reduce the occurrence of inflammation from the perspective of gross pathology, thus protecting the body.

**Conclusion:**

Leptin pretreatment can reduce MIRI injury, and the protective mechanism may be that leptin upregulates PI3K-AKT-NF*κ*B expression in myocardial tissue to reduce inflammation and promote repair of I/R injury.

## 1. Introduction

Cardiovascular disease is the deadliest disease in the world. Ischaemic heart disease is estimated by the World Health Organization to be the leading cause of death globally, causing more than 9 million deaths every year [1]. Coronary heart disease (CHD) remains a major cause of mortality and morbidity in developed countries. Over the past 40 years, the global death rate from coronary heart disease has decreased; however, about a third or more of deaths from coronary heart disease occur in people over 35 [2]. Myocardial ischemic reperfusion injury (MIRI) is central to the pathology of the most common cardiovascular diseases [1].

Ischemia/reperfusion (I/R) injury is cardiac tissue injury caused by blood flow recovery during repair [3]. I/R injury includes ion accumulation, mitochondrial membrane damage, formation of reactive oxygen species (ROS), disorder of nitric oxide (NO) metabolism, endothelial dysfunction, platelet aggregation, immune activation, apoptosis, and autophagy [4]. However, the molecular mechanism of myocardial I/R injury is not completely clear. Reperfusion of ischemic myocardium leads to reversible systolic dysfunction. Although restoring the blood flow of ischemic organs can prevent irreversible cell damage, reperfusion only once causes more tissue damage than ischemia [5].

Leptin is one of the neuropeptide hormones secreted by adipose tissue of the body, and its biological activity is mainly manifested in the maintenance of energy balance and fat metabolism of the body [6]. Leptin receptor is a single/transmembrane cell surface receptor, and leptin plays its physiological role by binding to the receptor on the cell membrane [7]. There is evidence that leptin has a cardioprotective effect in part by acting as an inflammatory suppressor in the heart muscle [8].

MIRI is the most common cause of death in clinical settings such as myocardial infarction, vascular surgery, and organ transplantation. MIRI is a very key and important problem in the clinical treatment of cardiac diseases. There is currently no definitive treatment for myocardial MIRI, even though heart-related mortality and morbidity have increased with advances in technology and science.

The significance of this study is to further explore the regulatory and protective effects of leptin on MIRI in rats through inflammatory signaling pathways at tissue molecular level and protein pathway. It provides experimental theoretical basis and new drug therapeutic target for the prevention and treatment of MIRI in clinic.

## 2. Materials and Methods

### 2.1. Animals and Ethics Statement

Adult female Sprague-Dawley (SD) rats weighing 250 ± 20 g were purchased from Shanghai University Animal Laboratory Center, reared in laboratory pathogen free conditions, 25 ± 2°C 12 : 12 h light-dark cycle and 50% ± 15% humidity. Animals have free access to regular pellet feed and tap water. All animals follow the internationally recognized ethics of the Shanghai University.

### 2.2. I/R Model Construction

Before surgery, the patient was fasted for 12 hours and given 30 mg/kg pentobarbital sodium solution by abdominal injection according to body weight for anesthesia. When the corneal reflex and pain sensation disappeared, a stable limb lead electrocardiogram was obtained. The blunt tip guide wire of the upper teeth of rats was lifted up to the tongue root, trachea intubation was performed, ventilators were connected, and positive pressure breathing was applied to provide respiratory function for the experimental rats during the operation.

The skin between the 2nd and 5th costals of the left chest was incised, the 2nd to 3rd intercostal muscles were incised, and the heart was fully exposed using a chest dilator. There is a ligation of the left anterior descending coronary artery. In this experiment, the ischemia time was 30 minutes, and the heart beat and electrocardiogram were closely observed 0-15 minutes after ligation.

Criteria for successful establishment of model: (1) the color of myocardium below ligation line and at apex of heart becomes gray white; (2) slow heart rate and weakened cardiac beat; (3) the ST segment of the limb lead of electrocardiogram was raised in the shape of a bow. After 30 minutes, the ligation line was cut with vascular scissors, and the heart was reperfused for 120 minutes.

In the sham group, the chest was also opened under anesthesia and ventilator, and ligation line was placed under the left anterior descending coronary artery, but no ligation was performed, and normal perfusion was performed for 150 minutes. After the operation, the head was cut off, and the ventricular blood and myocardial tissue were collected for storage. The leptin was given through the blood. The leptin low-dose intervention group and high-dose intervention group were 40 *μ*g/kg and 80 *μ*g/kg, respectively.

### 2.3. Assessment of Myocardial Infarction Area

After the animals were killed, the hearts were stripped immediately and washed repeatedly with precooled normal saline to wash away the residual blood. Then, put the sample heart in the refrigerator at -20°C and freeze it for about 15 minutes. Under the ligation line, cut the heart into 2 mm thick slices with a blade perpendicular to the long axis of the heart. The heart slices were placed in 1% TTC dye solution and incubated at 37°C for 10 minutes. After incubation, the heart slices were fixed in 4% paraformaldehyde for 24 hours. After fixation, suck the surface liquid of the heart slice with filter paper and take pictures. Red indicates noninfarct area, and gray indicates infarct area image. Calculate the myocardial infarction ratio [(infarct area/whole heart area) × Percentage].

### 2.4. Measurement of Left Ventricular-Related Data

Heparinized experimental animals were connected to medical arterial manometry tube with 15 g injection needle at 120 min of myocardial reperfusion. The injection needle was directly inserted into the left ventricle from the apex of the heart, the ventricular capsule of the manometric catheter was sent to the left ventricle, the other end of the arterial manometric catheter was connected with a biological recorder to measure its pressure, and the LVSP, LVEDP, dp/dt max, and -dp/dt max of rats were measured. The experiment was repeated three times.

### 2.5. Determination of Blood Myocardial Enzymes

Take the blood samples out of the -20°C refrigerator, thaw at room temperature, centrifuge for about 20 minutes (3000 rpm), and take the supernatant. The activities of lactate dehydrogenase (LDH) and creatine kinase (CK) in serum supernatant were measured by commercial kit to evaluate the degree of heart injury.

### 2.6. Histopathological Observation

Heart tissue was separated, embedded in paraffin, and stained with HE: after the tissue sections were baked in a 60°C oven for 2 h, the paraffin sections were dewaxed, hydrated and dehydrated, washed with tap water, and drained. Stained with hematoxylin and eosin. Then, slice and dehydrate. Put xylene in for 10 minutes, blow dry in the fume hood, seal the sheet with neutral gum, and dry at room temperature. The morphological changes of myocardium were observed by optical microscope by different observers for three times.

### 2.7. Western Blot

The heart tissue was isolated and homogenized after liquid nitrogen freezing, and the protein was extracted. The protein concentration of the cell was detected by BCA. An appropriate amount of protein concentrate was taken, the absorbance value was detected, and the sample concentration was calculated. After adjusting the concentration of protein, the membrane was separated by 12%SDS-PAGE electrophoresis and transferred to PVDF membrane after ionization. Sealed with 5% skim milk powder and successively added sheep anti-rabbit IgG labeled with primary antibody and horseradish peroxidase. The photoluminescence solution was added to develop and exposure, the image was obtained by gel imaging system, and the gray value of the strip was analyzed by Quantity One 4.4 software.

### 2.8. Statistical Analysis

All measurement data are expressed as mean ± standard deviation (*X* ± *S*). SPSS17.0 statistical software is used to analyze the data, and GraphPad Prism 6.0 is used to draw the data. Single-factor analysis of variance (ANOVA) was used for comparison among groups, and LSD-*t* test was used for pairwise comparison. *p* < 0.05 was considered statistically significant.

## 3. Results

### 3.1. Effects of Leptin on Cardiac Hemodynamics

**Figure 1:**
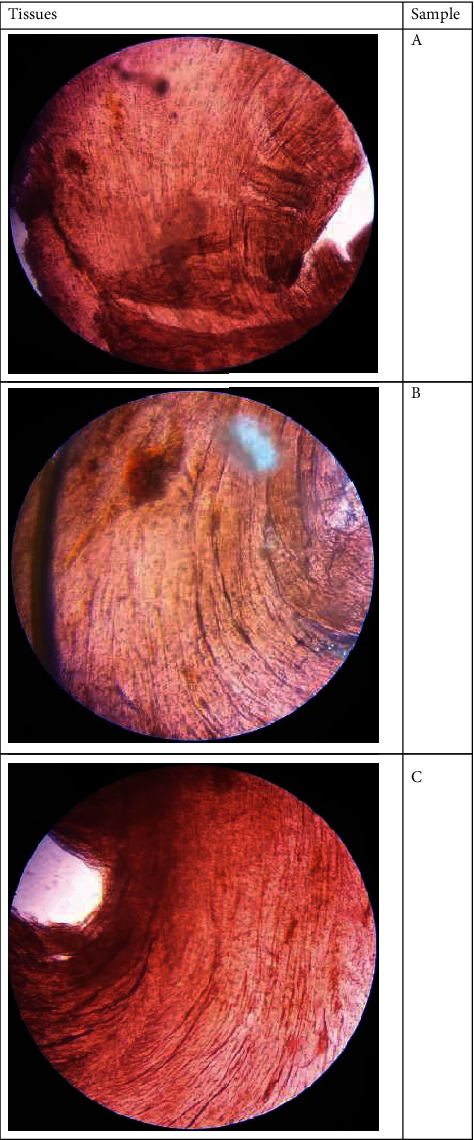
Tissue Samples of smooth muscles in off-pump coronary artery bypass grafting patients.

After 30 min of ischemia-reperfusion, the hemodynamic indexes of isolated heart in I/R group were significantly changed compared with the blank group, in which the maximum rate of increase of left ventricular pressure (+dp/dt max) and the maximum rate of decrease of left ventricular pressure (-dp/dt max) were significantly decreased (*p* < 0.05). The left ventricular end-diastolic pressure (LVEDP) was significantly increased (*p* < 0.05), while the left indoor pressure (LVSP) was significantly decreased (*p* < 0.05), indicating that the experimental modeling was successful. The above indexes were significantly improved after high- and low-dose preperfusion of leptin (*p* < 0.05). The results suggest that leptin can alleviate ischemia-reperfusion injury in isolated rat heart and improve the systolic function of damaged myocardium (Figure 1).

### 3.2. Effects of Leptin on Myocardial Enzymes LDH and CK

**Figure 2:**
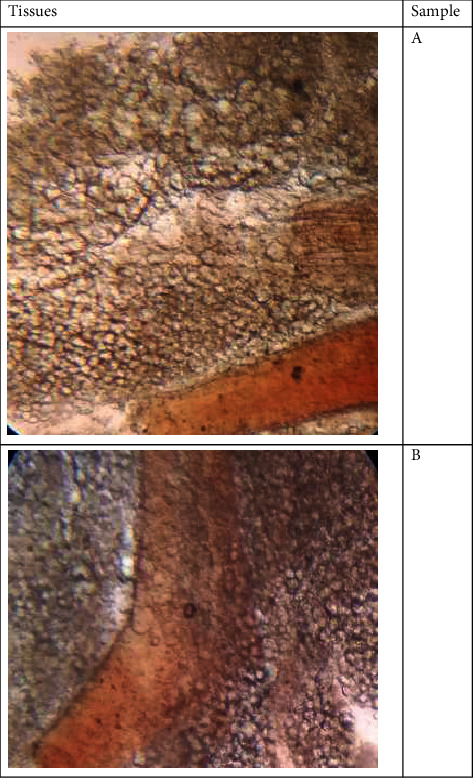
Fibrosis reported in patients suffering from acute kidney injury.

As shown in Figure 2, compared with the blank operation group, serum LDH and CK levels in I/R group were significantly increased (*p* < 0.05), while serum LDH and CK levels in leptin treatment group were significantly decreased (*p* < 0.05). LDH and CK levels of 80 mg/kg leptin consumption group were significantly lower than 40 mg/kg leptin consumption group (*p* < 0.05).

### 3.3. Effects of Leptin on Serum and Cardiac Inflammatory Cytokines

**Figure 3:**
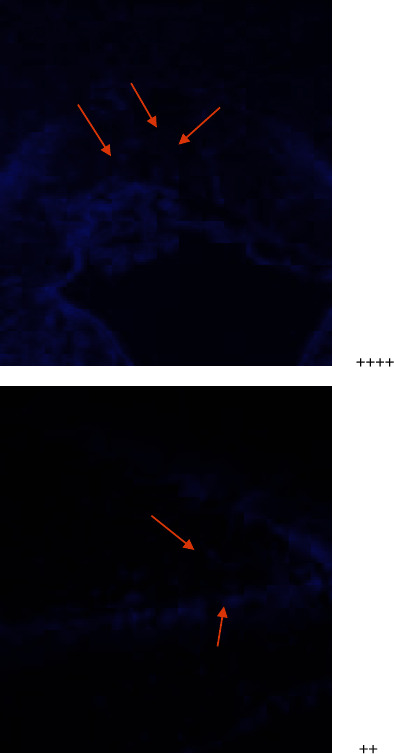


As shown in Figure 3, serum and heart levels of TNF-*α* and IL-6 were increased in the I/R group compared with the sham group (*p* < 0.05). The levels of TNF-*α* and IL-6 in serum and heart were significantly decreased in the leptin group compared with the I/R group (*p* < 0.05). Using 80 mg/kg leptin can make serum level of IL-6 and heart levels of IL-6 and TNF-*α* significantly lower than 40 mg/kg (*p* < 0.05).

### 3.4. Observation on Pathological Morphology of Myocardial Tissue

**Figure 4:**
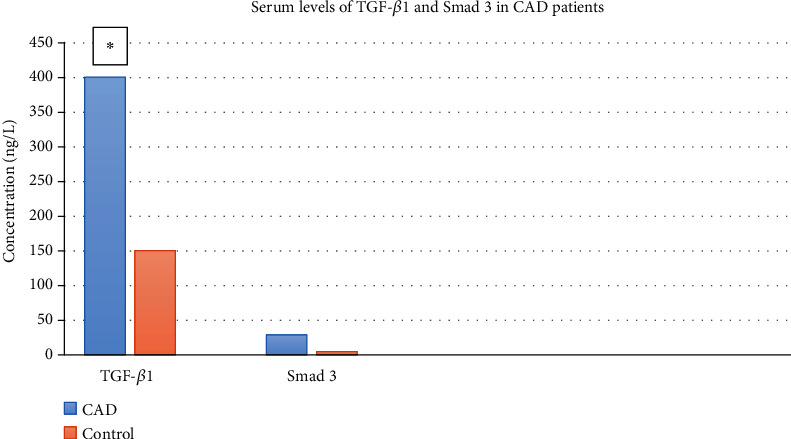
Serum levels of TGF-1 and Smad 3 in CAD patients with and without CAD0

In the I/R group, myocardial cell edema, erythrocyte exudation in the interstitium, a large number of inflammatory cell infiltration, and myocardial fiber arrangement disorder were observed. The tissue morphology of blank control group was basically normal with a little erythrocyte infiltration. In the low-dose leptin group, there was little edema of myocardial cells, inflammatory cell infiltration, and tissue interstitial widening, but there was little erythrocyte exudation, and myocardial fibers were arranged. In the high-dose leptin group, myocardial cell edema was not obvious, inflammatory cells were few, a small amount of red blood cell exudation was observed, and myocardial fiber changes were not obvious (Figure 4).

The lesions were divided into five grades according to the size, number, and severity of the lesions. Grade 0: no lesions; grade I: subendocardial focal lesions; grade II: extensive focal lesions of myocardium, swelling, and rupture of muscle fibers; grade III: extensive myocardial fusion lesions; grade IV: myocardial degeneration and necrosis. The grading results of pathological sections in this experiment are shown in Figure 4. Compared with the sham group, the grading results of I/R group were significantly higher (*p* < 0.01); the grading result of leptin group was significantly lower than that of I/R group (*p* < 0.05). The grading result of leptin 80 group was significantly lower than that of leptin 40 group (*p* < 0.05).

### 3.5. The Expression of PI3K, AKT, and NF*κ*B in Myocardial Tissue

**Figure 5:**
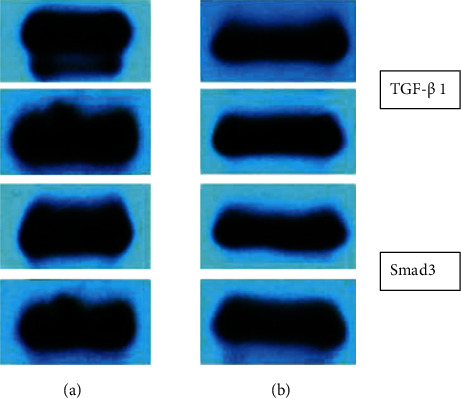
Expressions of TGF- 1 and Smad3 in both control and acute kidney injury group

Compared with blank group, the expression of PI3K, AKT, and NF*κ*B protein in I/R group decreased, while the expression of PI3K, AKT, and NF*κ*B increased in leptin group, while the expression of AKT and NF*κ*B increased most obviously in leptin-40 group. The expression of PI3K in leptin-80 group was most significantly upregulated, with statistical significance (*p* < 0.05). The PI3K expression of leptin-40 and leptin-80 was statistically significant (*p* < 0.05) (Figure 5).

## 4. Discussion

Acute myocardial infarction remains the leading cause of death and disability worldwide. The obvious remedy for ischemia is to restore blood flow as soon as possible. However, the restoration of oxygenated blood can cause further damage beyond the initial ischemic injury, which is known as ischemia/reperfusion (I/R) injury [9]. Timely reperfusion is essential to save viable myocardium during acute myocardial ischemia. However, this intervention is challenged by reperfusion injury. Researchers have been working to mitigate reperfusion injury and improve long-term outcomes from different aspects of infarction [10, 11].

Leptin is a peptide hormone synthesized by white adipose tissue. The leptin gene (LEP or ob) is located on chromosome 7q31.3 [12]. Mature proteins consist of 146 amino acids and are produced by mRNA directed protein synthesis [13]. Studies have shown that normal physiological levels of leptin are indeed essential for maintaining proper cardiovascular function. Obesity-related hyperleptinemia is an important biomarker for predicting cardiovascular outcomes, suggesting that leptin plays a critical role in obesity-related cardiovascular disease [14]. Leptin deficiency or resistance is related to the imbalance of cytokine production, increased susceptibility to infection, autoimmune diseases, malnutrition, and inflammatory response [15]. Studies have shown that compared with wild-type (WT) FVB/N control mice, leptin is a long-lived transgene overexpressing leptin *α*MUPA mice were endowed with resistance to cardiac ischemic injury. Exogenous leptin pretreatment significantly reduced TNF after hypoxia *α* and IL-1 *β*. At the same time, leptin enhances the expression of all other genes and reduces the level of ROS. Leptin affects the gene expression of cardiomyocytes under hypoxia, which can reduce inflammation and oxidative stress [16].

More and more data have proved that the activation of PI3K/AKT signaling pathway is the key molecular mechanism for ischemia-reperfusion to play a multieffect under various pathological conditions [17]. The activity of PI3K/AKT pathway has a variety of effects on MIRI [18, 19]. PI3K and its downstream target serine/threonine kinase AKT belong to a conserved signal transduction enzyme family. The activation of PI3K/AKT pathway is considered to be an endogenous regulatory mechanism, which can promote cell survival in response to harmful external stimuli [20]. The occurrence of Miri involves many factors, and the damage to cells caused by inflammatory response is an important pathological mechanism [21]. NF*κ*B is an important inflammatory transcription factor, which widely exists in eukaryotic cells and participates in the transcriptional regulation of many apoptosis-related genes. Previous studies reported that NF*κ*B is essential in cardiovascular disease and is activated in the early pathogenesis of ischemia [22].

In this study, it was found experimentally that leptin can improve the hemodynamics of cardiac ischemia-reperfusion rats, improve the expression of myocardial enzymology, reduce the release of cardiac and serum inflammatory factors, and reduce the occurrence of inflammation from the perspective of gross pathology, thus protecting the body. Its protective pathway may play a role through the PI3K-AKT-NF*κ*B pathway and participate in the regulation of MIRI.

## Data Availability

The data used to support this study is available from the corresponding author upon request.
